# The Prognostic Role of SOCS3 and A20 in Human Cholangiocarcinoma

**DOI:** 10.1371/journal.pone.0141165

**Published:** 2015-10-20

**Authors:** Yimin Wang, Ming Wan, Qingxin Zhou, Hao Wang, Zhidong Wang, Xiangyu Zhong, Lei Zhang, Sheng Tai, Yunfu Cui

**Affiliations:** 1 Department of Hepatopancreatobiliary Surgery, Second Affiliated Hospital of Harbin Medical University, Harbin, Heilongjiang, P. R. China; 2 Key Laboratory of Myocardial Ischemia Mechanism and Treatment Ministry of Education, Harbin, Heilongjiang, P. R. China; INRS, CANADA

## Abstract

As an antagonist of the JAK/STAT pathway, suppressor of cytokine signaling 3 (SOCS3) plays an integral role in shaping the inflammatory environment, tumorigenesis and disease progression in cholangiocarcinoma (CCA); however, its prognostic significance remains unclear. Although tumor necrosis factor α-induced protein 3 (TNFAIP3, also known as A20) can decrease SOCS3 expression and is involved in the regulation of tumorigenesis in certain malignancies, its role in CCA remains unknown. In this study, we investigated the expression of SOCS3 and A20 in human CCA tissues to assess the prognostic significance of these proteins. The expression of SOCS3 and A20 was initially detected by western blot in 22 cases of freshly frozen CCA tumors with corresponding peritumoral tissues and 22 control normal bile duct tissues. Then, these proteins were investigated in 86 CCA patients by immunohistochemistry (IHC) and were evaluated for their association with clinicopathological parameters in human CCA. The results indicated that SOCS3 expression was significantly lower in CCA tumor tissues than in corresponding peritumoral biliary tissues and normal bile duct tissues. Conversely, A20 was overexpressed in CCA tissues. Thus, an inverse correlation between the expression of SOCS3 and A20 was discovered. Furthermore, patients with low SOCS3 expression or high A20 expression showed a dramatically lower overall survival rate. These proteins were both associated with CCA lymph node metastasis, postoperative recurrence and overall survival rate. However, only A20 showed a significant association with the tumor node metastasis (TNM) stage, while SOCS3 showed a significant association with tumor differentiation. Multivariate Cox analysis revealed that SOCS3 and A20 were independent prognostic indicators for overall survival in CCA. Thus, our study demonstrated that SOCS3 and A20 represent novel prognostic factors for human CCA.

## Introduction

Cholangiocarcinoma (CCA) is the second most common primary hepatobiliary cancer, arising from the biliary tree with characteristic cholangiocyte differentiation, and epidemiological studies have shown that the incidence of CCA is increasing worldwide [1–4]. Complete surgical resection is still the most preferred and only possible curative treatment for this fatal disease [5]. Unfortunately, most patients are diagnosed at an unresectable stage, where the prognosis of CCA is notoriously poor [6]. Thus, the discovery of effective biomarkers for prognosis, with a view to define the molecular mechanisms underlying CCA tumor development and progression, remains an urgent need.

Chronic biliary inflammation is a confirmed risk factor for CCA, which thus represents a classic model disease to study the relationship between chronic inflammation and the initiation and progression of cancers [7, 8]. The JAK/STAT pathway has been shown to play an integral role in shaping the inflammatory environment of CCA and other cancers [9, 10]. The JAK/STAT pathway regulates a variety of vital processes including innate and adaptive immune function and embryonic development, as well as cell proliferation, differentiation and apoptosis [11], and its key role in regulating human biliary epithelial cell migration has been demonstrated in our prior studies [12].

The suppressors of cytokine signaling (SOCS) proteins function as cytokine signaling inhibitors of the JAK/STAT pathway. Thus far, there have been eight SOCS proteins identified, and these family members possess similar structures but differential mechanisms for inhibiting the JAK/STAT pathway. As part of a classical feedback loop, SOCS3 expression competes with STAT activation by inhibiting its phosphorylation, which is mediated by the stimulation of cytokines or growth factors. Moreover, SOCS3 binds to cytokine receptors that contain JAK-proximal sites, leading to JAK inhibition [13, 14]. Additionally, SOCS3 acts as a negative regulator in the activation of STAT3 and chronic inflammatory processes [15]. Loss of SOCS3 expression has been reported in a variety of malignancies due to epigenetic mechanisms, mostly promoter methylation [16–20]. In CCA, this mechanism was confirmed in an earlier study as well [21]. In liver, lung, and squamous head and neck cancer, as well as a number of hematological malignancies, SOCS3 functions as a classical tumor suppressor [21]. Our recent studies suggested that enhanced expression of SOCS3 could reduce tumor metastasis, the expression of epithelial-to-mesenchymal transition (EMT) markers and STAT3 activation in the absence of interleukin-6 (IL-6) stimulation in CCA cell lines [22]. Very little is known about SOCS3 expression in human CCA tissue and whether SOCS3 may serve as a novel prognostic biomarker for CCA patients.

A20, also known as tumor necrosis factor α-induced protein 3 (TNFAIP3), is a zinc-finger protein that plays a pivotal negative role in the regulation of inflammation and immunity [23]. It was recently discovered in liver regeneration and repair that A20 can increase JAK/STAT3 pro-proliferative signals by decreasing SOCS3 expression, most likely in a miR203-dependent manner; furthermore, A20 can reduce the levels of the cell cycle inhibitor p21 [24]. Moreover, A20 plays an oncogenic role, as indicated by A20 overexpression in several malignancies, such as undifferentiated nasopharyngeal carcinoma, poorly differentiated head and neck squamous cell carcinoma [25], glioma [26, 27], glioblastoma [28], inflammatory breast cancer [29], and hepatocellular carcinoma [30]. Overexpression of A20 in breast cancer cells contributes to resistance to TNFα and tamoxifen, indicating that A20 induces chemoresistance and survival [31]. Although the data above suggest a tumorigenic role for A20 overexpression, loss of A20 function is associated with multiple lymphomas, including B-cell lymphoma [32, 33], non-Hodgkin lymphoma [34], and Hodgkin lymphoma [35]. Taken together, these results have led several investigators to posit that A20 plays a contextual role in tumor biology that may be tissue-type-dependent [27]. However, the role of A20 expression in human CCA remains unclear but pathophysiologically important.

The aim of the present study was to investigate the status of SOCS3 and A20 expression in human CCA tissue by western blot and immunohistochemistry (IHC), as well as their correlation with clinicopathological parameters. Survival analyses were performed to evaluate the prognostic relevance of these two biomarkers. Therefore, this study has momentous implications for further elucidating the molecular mechanisms of human CCA. Our data suggest that SOCS3 and A20 may serve as novel prognostic biomarkers for CCA patients.

## Patients and Methods

### Ethics statement

This study and all involved protocols were approved by the ethical committee of the Second Affiliated Hospital of Harbin Medical University. Specimens were obtained with written informed consent, which was signed by patients or their next of kin on behalf of the patients involved in this study.

### Fresh-frozen tissue samples

Fresh tumor and corresponding peritumoral biliary tissues were collected from 22 CCA patients who underwent curative surgery and whose CCA was pathologically confirmed intraoperatively between November 2013 and October 2014 at the Second Affiliated Hospital of Harbin Medical University, Heilongjiang, China. Pathologists used frozen tissue examination during the operation to confirm CCA according to following criteria: tumor tissues contained at least 70% tumor cells, while the peritumoral tissues contained no tumor cells. In addition to the CCA specimens, we also obtained 22 normal biliary duct specimens as controls, which were collected from patients undergoing hepatectomy or pancreatoduodenectomy for non-tumor-related diseases (giant hepatic hemangioma, traumatic rupture of duodenum and pancreas trauma), where the selected biliary duct tissues were disease-free and no local inflammation was detected under gross and microscopic observation. All selected tissues were preserved in liquid nitrogen.

### Western blot analysis

To determine the expression levels of SOCS3 and A20 in CCA, western blot analyses were carried out as previously described [36] for the 22 cases of CCA where freshly frozen tumors and their corresponding peritumoral tissues were available. A 100 mg sample was resected from each pair of tumor and peritumoral tissues and washed 3 times with ice-cold phosphate-buffered saline (PBS). The sample tissues were ruptured with RIPA buffer (50 mM Tris-HCl, pH 7.4, 150 mM NaCl, 1% NP-40, 0.5% sodium deoxycholate, 0.1% SDS) containing 100 mM phenylmethylsulfonyl fluoride (PMSF; catalog number P0013B, Beyotime Institute of Biotechnology, China) on ice for 30 min. The tissue extracts were centrifuged for 30 min at 14,000×g and 4°C, and the supernatants were collected. The tissue sample supernatants containing equal amounts of protein were resolved by 8% SDS-polyacrylamide gel electrophoresis. Then, the proteins were transferred to polyvinylidene difluoride (PVDF) membranes. These membranes were blocked with 5% skim milk in Tris-buffered saline containing 0.1% Tween-20 (TBST) for 1 h at room temperature and probed overnight at 4°C with the appropriate primary antibodies [anti-SOCS3 rabbit polyclonal antibody (catalog number sc-9023, Santa Cruz Biotechnology, Inc.), anti-A20 mouse monoclonal antibody (catalog number sc-166692, Santa Cruz Biotechnology, Inc.) and anti-β-actin mouse monoclonal antibody (Zhongshan Goldenbridge Biotechnology Co. Ltd., China), all at a dilution of 1:1,000]. Then, the membranes were rinsed with TBST before a 1-h incubation with horseradish peroxidase (HRP)-conjugated secondary antibodies [goat anti-rabbit IgG (H+L)-labeled secondary antibody (catalog number A0208, Beyotime Institute of Biotechnology, China) and goat anti-mouse IgG (H+L)-labeled secondary antibody (catalog number A0192, Beyotime Institute of Biotechnology, China), both at a dilution of 1:1500]. Membranes were washed in TBST solution with 0.5% Tween-20 before enhanced chemiluminescence (ECL) detection with BeyoECL Plus (catalog number P0018, Beyotime Institute of Biotechnology, China). After exposure on X-ray film, protein band densitometry was quantified using the Gel Image Analysis System (Tanon, Shanghai, China), and the antibody against β-actin was used for normalization as previously described [37, 38]. Concurrently, the expression levels of SOCS3 and A20 in the 22 cases of normal biliary duct tissues were detected by western blot analysis according to the above method.

### Patients and clinical data

A total of 109 patients who underwent curative surgery, whose CCA (adenocarcinoma subtype) was later pathologically confirmed and who did not have any other malignancy between December 2009 and March 2012 at the Second Affiliated Hospital of Harbin Medical University were retrospectively reviewed. No patients received chemotherapy or radiotherapy before or after surgery in this study. Patients who died of unrelated diseases or within one month after surgery were excluded, leaving 86 patients eligible for this study. These patients consisted of 54 males and 32 females with a median age of 61.5 years (range of 42–83 years). The pathological data for CCA were evaluated according to the 7^th^ edition of the American Joint Committee on Cancer (AJCC). Other clinical data from the patients, such as tumor site, differentiation, histological patterns, lymph node metastasis, vascular invasion, tumor node metastasis (TNM) stage, postoperative recurrence and 3-year survival, were obtained from pathology reports and medical records, along with preoperative serum CEA (carcinoembryonic antigen) and CA19-9 (carbohydrate antigen 19–9) levels and HBV (hepatitis B virus) infection status. Overall survival was calculated as the interval from the date of surgery to death or the date of the latest follow-up for the living patients. Each CCA patient was followed until March 2015 or their date of death, and the median follow-up period was 23 months (ranging from 2 to 48 months) (S1 Table).

Curative surgery for CCA was performed as complete resection of the cancer mass (without a positive surgical margin according to pathological examination), dissection of the regional lymphonodi, and removal of the cancer embolus in the regional vessels and biliary ducts. The CCA specimens were obtained from regions close to the cancer margin. All surgical specimens were made into formalin-fixed paraffin-embedded (FFPE) blocks by pathologists and stored in the pathology department.

### IHC

IHC was performed as previously described [4]. FFPE blocks were cut at a thickness of 4 μm, placed and fixed on positively charged slides, and heated at 55°C for 30 min. Then, the slides were deparaffinized in xylene and subsequently hydrated in a graded ethanol solution. The deparaffinized slides were stained with hematoxylin-eosin to identify typical FFPE blocks with tumor morphology. Selected FFPE blocks were cut into serial slides at 4-μm intervals for assessment. All slides were deparaffinized and rehydrated as mentioned above. Then, the slides were incubated with 3% hydrogen peroxide in PBS at room temperature for 20 min to quench endogenous peroxidase activity. After rinsing with PBS, antigen retrieval was carried out in citrate buffer (pH 6.0) for 15 min in a microwave oven. The slides were then incubated in normal goat serum for 30 min at 37°C to block nonspecific binding, followed by incubation with anti-SOCS3 rabbit polyclonal antibody (catalog number sc-9023, Santa Cruz Biotechnology, Inc.) or anti-A20 mouse monoclonal antibody (catalog number sc-166692, Santa Cruz Biotechnology, Inc.) at a dilution of 1:200 for 30 min at room temperature. Concurrently, PBS was applied instead of the primary antibodies for unstained slides as a negative control. Then, the slides were rinsed 3 times with PBS, followed by incubation with secondary antibody (biotin conjugated goat anti-rabbit or goat anti-mouse immunoglobulin, catalog numbers sc-2040 and sc-2039, Santa Cruz Biotechnology, Inc.) for 30 min. After washing with PBS, the slides were incubated with HRP-streptavidin reagent for 45 min. Diaminobenzidine (DAB) and hematoxylin were utilized for chromogenic detection and counterstaining on all slides.

### Assessment of IHC variables

The assessment of IHC variables was performed as described previously [39] with slight modifications. Semi-quantitative expression levels were based on the staining intensity of SOCS3/A20 and the distribution of positive tumor cells. The chromogenic reaction of SOCS3/A20 was classified as four grades by staining intensity using a scale of 0–3, where 0 corresponded to negative staining and 3 to strong staining. The percentage of SOCS3/A20 staining in five random non-overlapping fields of each slide at a final magnification of 400× was also classified by grade as follows: 0 (<25%), 1 (5–25%), 2 (26–50%), 3 (51–75%), and 4 (76–100%). The intensity and percentage scores were multiplied to obtain the final staining score [37]. The staining pattern for all slides was defined as follows: a final staining score of 6–12 was defined as high expression, while scores less than 6 were defined as low expression. Furthermore, each slide was blindly evaluated by two experienced independent pathologists unaware of the clinical data and follow-up information. The final staining scores from the two pathologists were compared, and any inconsistent scores were managed by reappraisal of the slide by both pathologists until a consistent score was reached. Images were obtained using an Olympus BX50 light microscope connected to a charge-coupled device (CCD) camera and the Image-Pro Plus 6.0 software (Media Cybernetics, Inc.).

### Statistical analysis

The data analyses were performed using the Statistical Package for the Social Sciences software (SPSS, version 19.0, Chicago, IL). The paired-samples t-test was used to compare the protein expression of SOCS3/A20 between CCA tumors and the corresponding peritumoral tissue samples. The relationship between SOCS3/A20 expression and the clinicopathological features at various stages of CCA progression was elucidated using a chi-square test. Kaplan-Meier analysis was applied to calculate survival curves. As mentioned above, the overall survival was calculated as the interval between the time of surgery and the time of death or the end of follow-up. The relationship between SOCS3 and A20 expression was evaluated by linear regression analysis and Spearman rank correlation. A univariate log-rank test was carried out to determine the significance of the clinicopathological parameters, and then the risk factors filtered by the log-rank test were further analyzed with the Cox hazards regression model to determine independent factors for prognosis. A *P* value less than 0.05 was considered statistically significant, with all *P* values based on two-sided statistical analysis.

## Results

### SOCS3 and A20 expression levels in CCA

**Fig 1:**
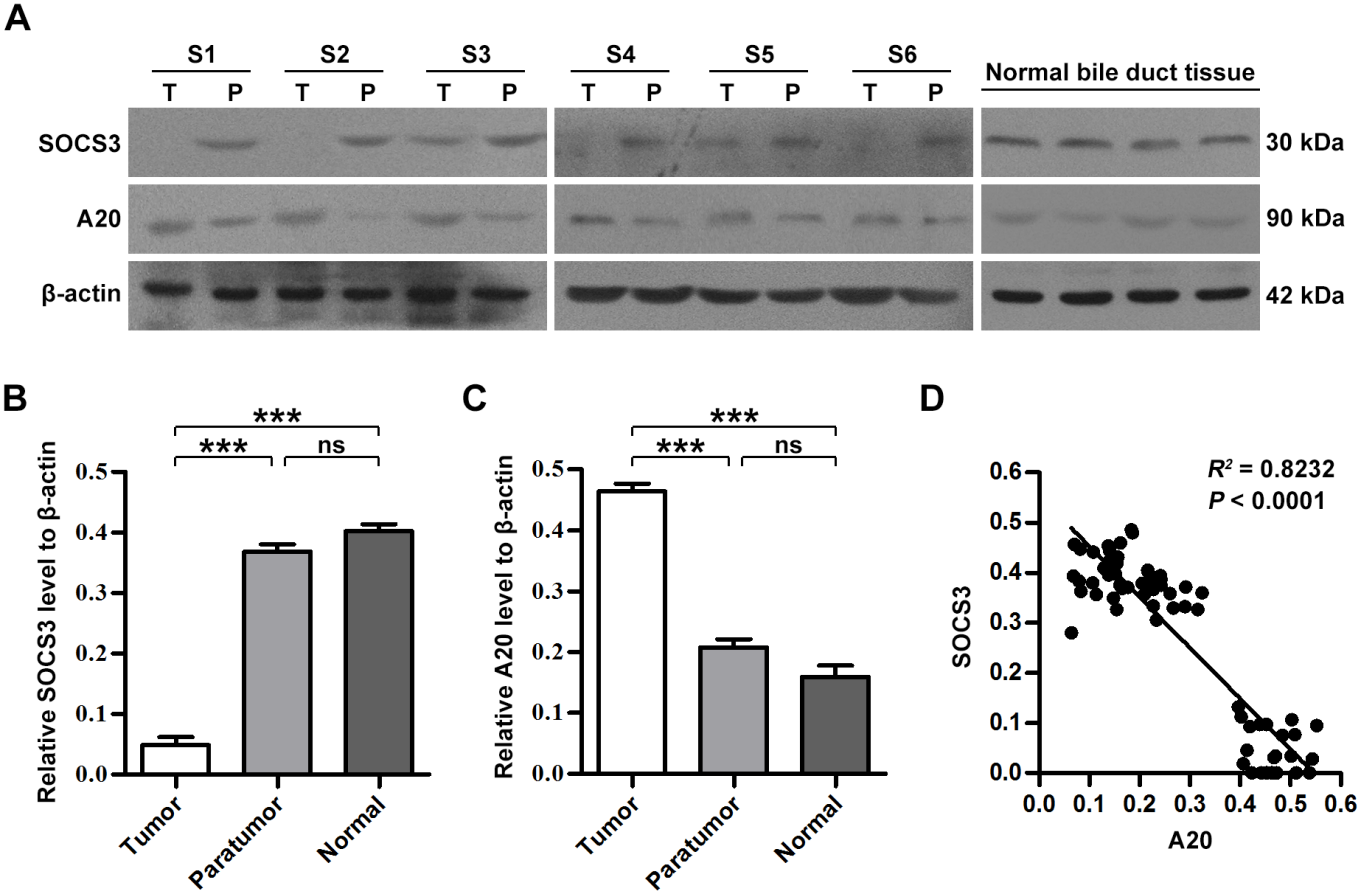
A20 and SOCS3 expression in CCA tissues. A. Western blot analysis of A20 and SOCS3 in CCA tumor tissues and normal biliary duct tissues. T, tumor tissue; P, peritumoral biliary tissue; N, normal biliary duct tissue. B. Expression index of SOCS3 protein. The SOCS3 protein expression level in T was significantly lower than that in P (*P <* 0.0001). C. Expression index of A20 protein. The A20 protein expression level in CCA was significantly higher than that in P (*P <* 0.0001). D. Linear regression analysis of A20 and SOCS3 expression in CCA tumor tissue and normal biliary duct tissues. Each point represents a tissue sample, with n = 66 cases (22 tumor, 22 paratumor, and 22 normal biliary duct tissues). The A20 level was inversely correlated with SOCS3 expression. A20 and SOCS3 protein levels were measured by expression index (the ratio of protein of interest to β-actin expression). ***: T compared with P or N, *P <* 0.05.

The protein expression levels of SOCS3 and A20 were first detected by western blot analysis in freshly frozen tumors and the corresponding peritumoral biliary tissues from 22 CCA cases. The SOCS3 signal was positive in only 27.27% (6 cases) of the tumor tissues, while it was positive in all corresponding peritumoral biliary tissues. Conversely, the A20 signal was positive in all tumor tissues and in 68.75% (15 cases) of the corresponding peritumoral biliary tissues (Fig 1A). The quantitative analysis of each protein band was standardized against β-actin expression in each sample, and the ratio of the protein of interest to β-actin expression was defined as the expression index. The protein expression level of SOCS3 in CCA tumor tissues was conspicuously lower than that in the corresponding peritumoral biliary tissues (7.56-fold on average, *P <* 0.0001), and it was also lower than that in normal biliary duct tissues (Fig 1B). In contrast, the protein expression level of A20 in CCA tumor tissues was conspicuously higher than that in the corresponding peritumoral biliary tissues (2.23-fold on average, *P <* 0.0001), and it was also significantly higher than the protein expression level of A20 in normal biliary duct tissues (Fig 1C). Linear regression analysis further revealed that the A20 level was inversely correlated with SOCS3 expression in CCA tumor tissues and normal biliary duct tissues (*R* ^*2*^ = 0.8232, *P <* 0.0001; Fig 1D).

**Fig 2:**
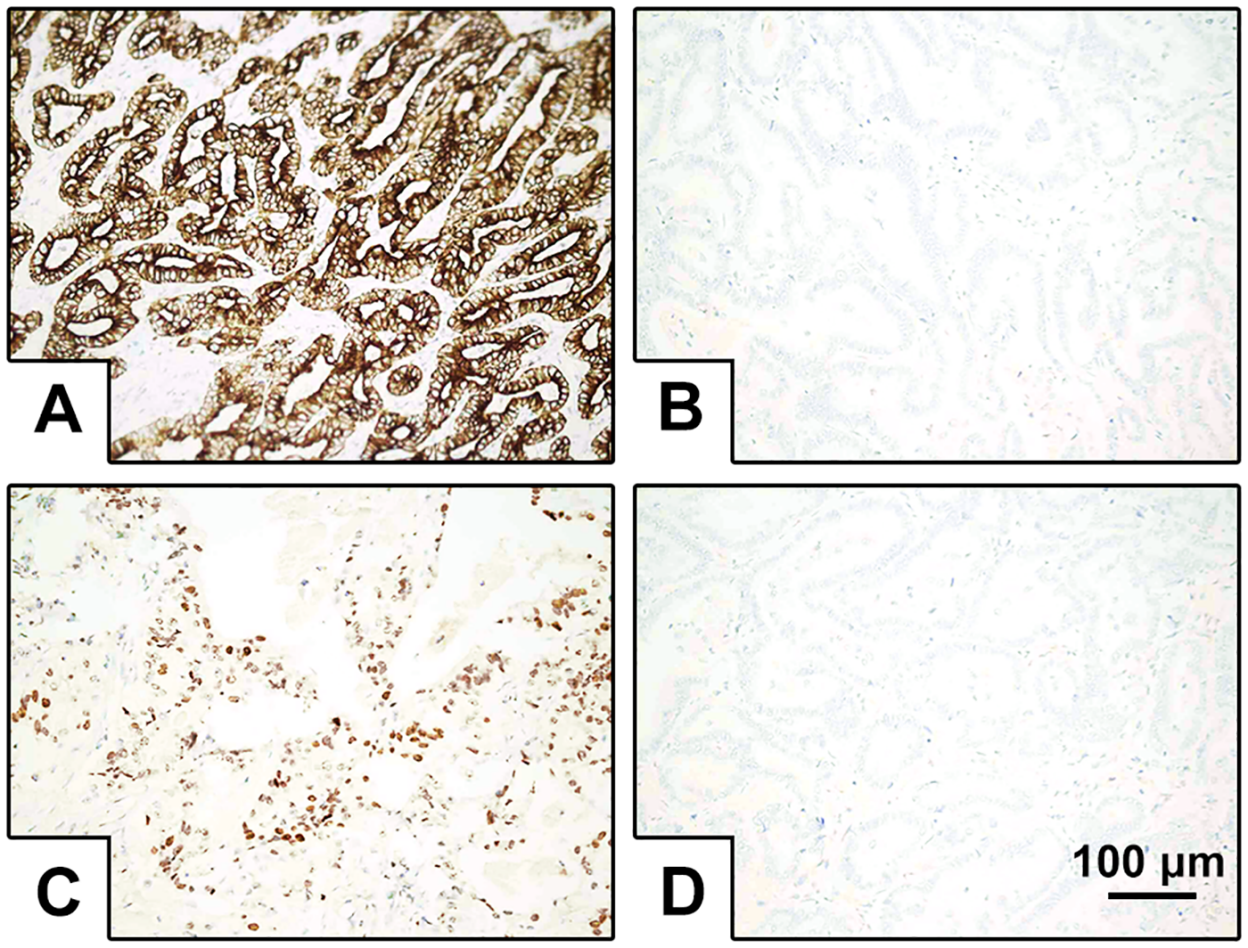
IHC staining for SOCS3 and A20 in human CCA. Original magnification 200×, scale bar = 100 μm. A. High SOCS3 expression was found predominately in the cytoplasm and cytomembrane in CCA tissues. B. Low SOCS3 expression in CCA tissues. C. High A20 expression was found in the nucleus. D. Low A20 expression in CCA tissues.

Next, the expression levels of SOCS3 and A20 were detected in tissue slides from 86 CCA cases by IHC. As mentioned above, SOCS3 expression was found predominately in the cytoplasm and cytomembrane, while A20 expression was detected in the nucleus (Fig 2, scale bar = 100 μm). According to the assessment criteria in this study, 62 (72.1%) of the 86 CCA specimens were classified as showing low expression for SOCS3, whereas 24 (27.9%) showed high expression. Conversely, for A20, 27 (31.4%) of the CCA specimens showed low expression, and 59 (69.6%) showed high expression. Consistent with the western blot analysis, the IHC results indicated decreased expression of SOCS3 and increased expression of A20 in CCA.

**Fig 3:**
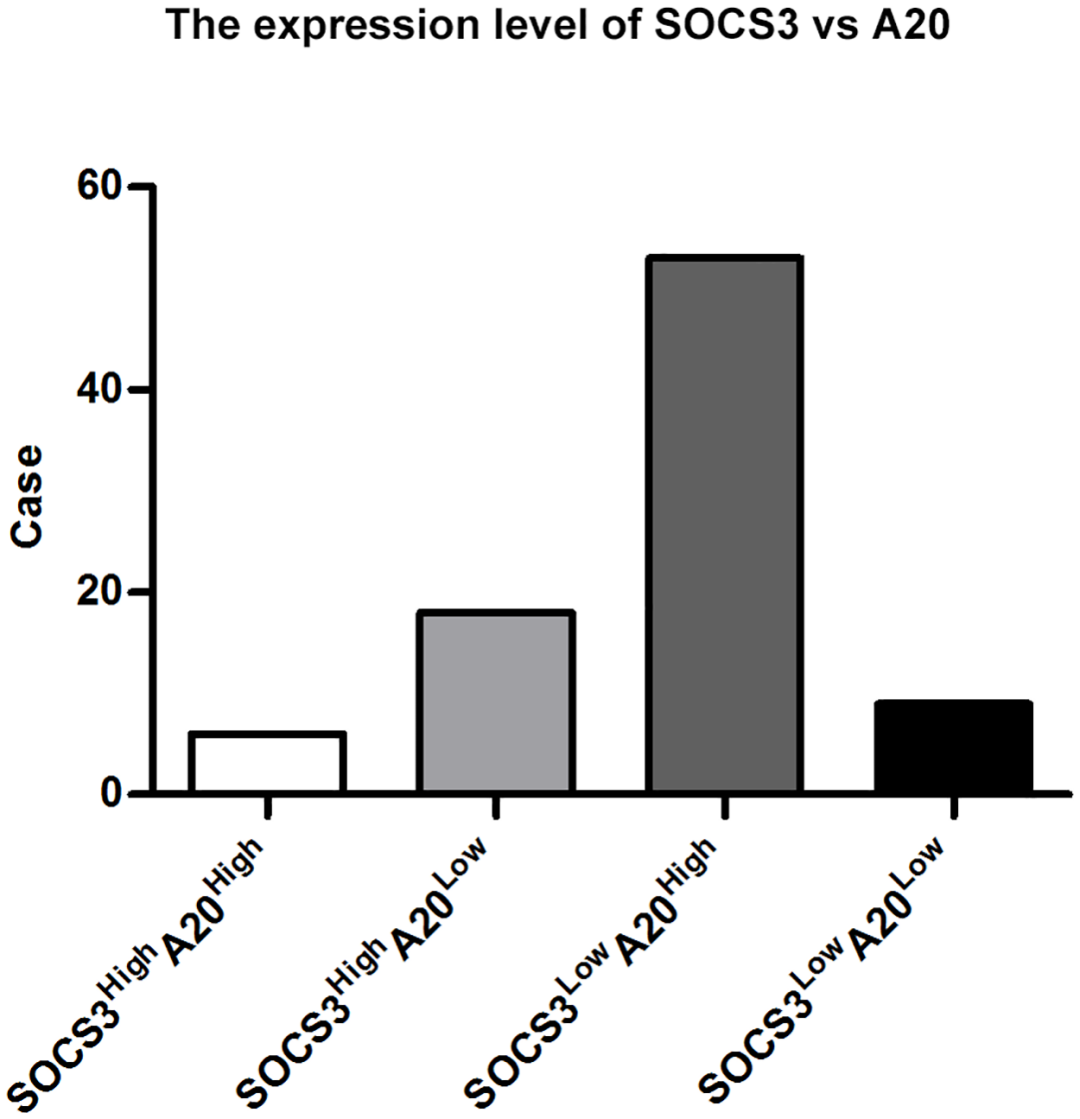
The inverse correlation between SOCS3 and A20. From left to right, the expression of SOCS3 is compared to that of A20 (n = 86) for high to high (n = 6), high to low (n = 18), low to high (n = 53) and low to low expression (n = 9) (r = -0.585, *P <* 0.0001).

To further identify the correlation between the expression of SOCS3 and A20, the 86 CCA specimens were assigned to four groups based on SOCS3 and A20 expression levels (Fig 3). In the 86 CCA samples, 6 (7.0%) showed high expression of both SOCS3 and A20, 9 (10.5%) showed low expression of both SOCS3 and A20, 53 (61.6%) showed high A20 and low SOCS3 expression, and 18 (20.9%) showed low A20 and high SOCS3 expression. Utilizing Spearman rank analysis, a significantly negative correlation between SOCS3 and A20 expression was verified (r = -0.585, *P <* 0.0001). Moreover, the data further confirmed the inverse relationship between the expression levels of SOCS3 and A20 in CCA.

### Patients’ characteristics

As shown in Table 1, 86 CCA patients were included in this study, and their average age was 61.6 years. The patients consisted of 32 males and 54 females. Among these patients, 18 had intrahepatic CCA, 33 had perihilar CCA, and 35 cases had distal CCA. In accordance with tumor differentiation, 16 cases were classified as Grade 1 (well differentiated), 38 cases as Grade 2 (moderately differentiated) and 32 cases as Grade 3 (poorly differentiated). Based on histological patterns, 76 cases were histologically classified as tubular adenocarcinoma, 6 cases as papillary adenocarcinoma and 4 cases as mucinous adenocarcinoma. A total of 51 patients suffered from lymph node metastasis, while 12 patients had vascular invasion. According to the 7^th^ edition of the AJCC classification, 12 cases were in stage I, 23 cases were in stage II, 32 cases were in stage III, 19 cases were in stage IV A, and stage IV B patients who were not amenable to surgery were excluded. Each CCA patient was followed until March 2015 or their date of death. The survival time in this study ranged from 2 to 48 months, with a median survival time of 23 months. A total of 52 patients died from recurrence during the follow-up period.

**Table 1:** SOCS3 and A20 expression status in relation to clinicopathologic features.

Clinicopathologic features	No. patients	SOCS3 expression level		*P*	A20 expression level		*P*
		High	Low		High	Low	
**Age (years)**							
<60	37	9	28	0.185	28	9	0.220
≥60	49	15	34		31	18	
**Gender**							
Male	54	13	41	0.303	40	14	0.156
Female	32	11	21		19	13	
**Tumor site**							
Intrahepatic	18	4	14	0.285	13	5	0.877
Perihilar	33	7	26		23	10	
Distal	35	13	22		23	12	
**Differentiation**							
Grade 1	16	10	6	**0.002**	7	9	0.160
Grade 2	38	9	29		27	11	
Grade 3	32	5	27		25	7	
**Histological patterns**							
Tubular adenocarcinoma	76	23	53	0.280	51	25	0.177
Papillary adenocarcinoma	4	1	3		2	2	
Mcinous adenocarcinoma	6	0	6		6	0	
**Lymph node metastasis**							
Positive	51	10	41	**0.038**	42	9	**0.001**
Negative	35	14	21		17	18	
**Vascular invasion**							
Positive	12	1	11	0.103	11	1	0.128
Negative	74	23	51		48	26	
**TNM stage**							
I	12	6	6	0.227	7	5	**0.001**
II	23	6	17		7	10	
III	32	9	23		30	2	
IVA	19	3	16		15	4	
**Postoperative recurrence**							
Present	50	9	41	**0.016**	39	11	**0.026**
Absent	36	15	21		20	16	
**Survival time**							
>3 years	34	14	20	**0.027**	19	15	**0.040**
≤3 years	52	10	42		40	12	
**Serum CEA**							
>5 ng/ml	51	12	39	0.275	36	15	0.632
≤5 ng/ml	35	12	23		23	12	
**Serum CA199**							
>37 U/ml	56	15	41	0.751	37	19	0.489
≤37 U/ml	30	9	21		22	8	
**HBV infection**							
Positive	42	11	31	0.729	30	12	0.581
Negative	44	13	31		29	15	

TNM stage, Tumor-Node-Metastasis stage; CEA, carcino embryonie antigen; CA199, carbohydrate antigen 19–9; HBV, Hepatitis B virus.

### SOCS3/A20 expression level and clinical data

To verify the relationship between SOCS3/A20 expression and major clinical data among the 86 CCA cases, chi-square tests were performed. When the SOCS3/A20 expression level was classified as high or low, no significant correlation was found between SOCS3/A20 expression and certain clinicopathological parameters, including gender, age, tumor site, histological patterns, vascular invasion, serum CEA and CA19-9 levels and HBV infection status (Table 1, all P values > 0.05). However, statistically significant correlations were found with lymph node metastasis, overall survival rate and postoperative recurrence (*P <* 0.05) for both SOCS3 and A20. Only A20 showed a statistically significant association with the TNM stage, while SOCS3 merely showed a significant association with tumor differentiation. These results revealed that low SOCS3 expression and high A20 expression in CCA correlated with the status of the disease.

**Fig 4:**
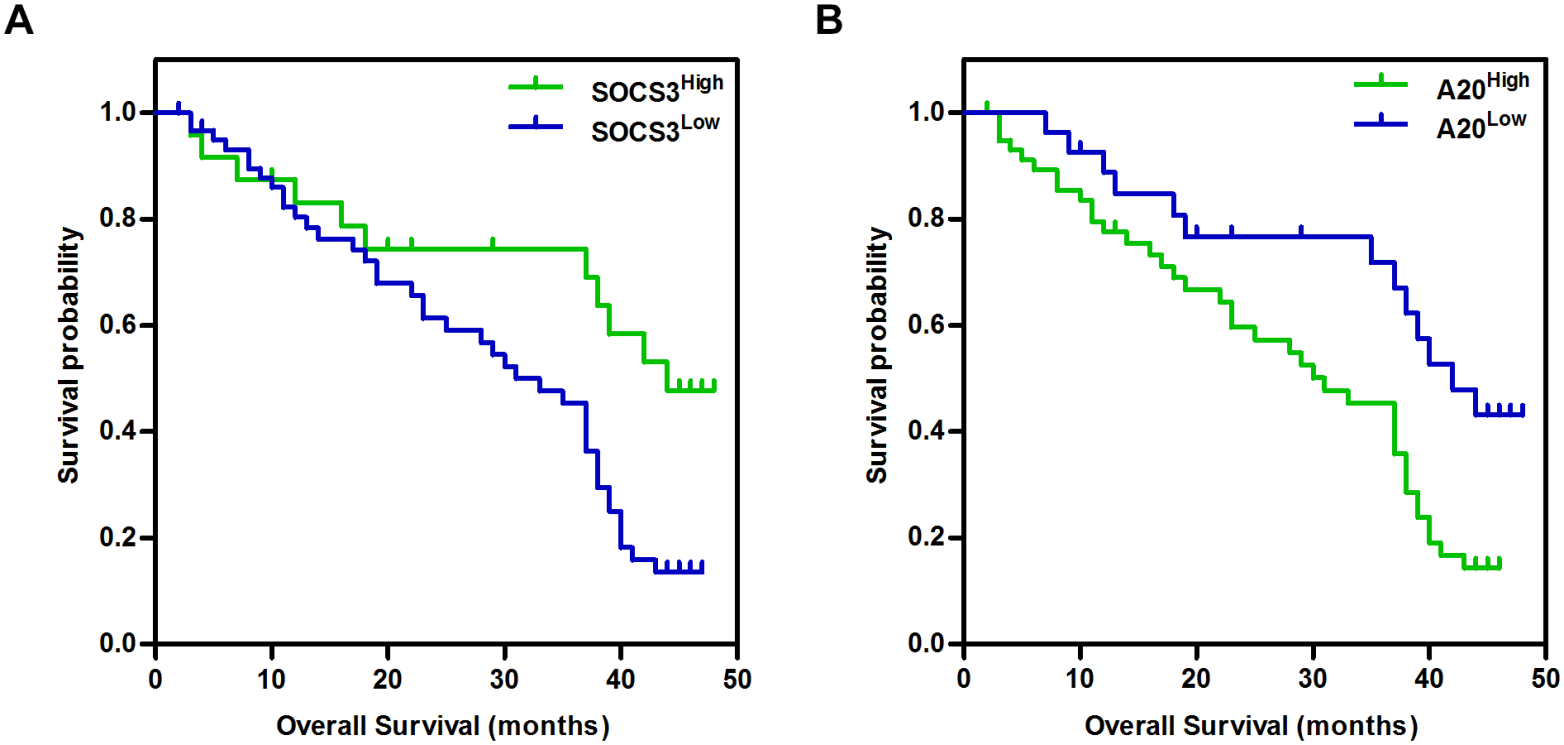
Kaplan-Meier curves of overall survival in CCA patients. A. The correlation with SOCS3 level. B. The correlation with A20 level.

To further investigate the correlation between SOCS3/A20 expression and overall survival in CCA, Kaplan-Meier analysis was applied to calculate survival curves. The results suggested that patients with low SOCS3 levels had a dramatically worse overall survival rate (*P* = 0.008, Fig 4A), while patients with high A20 levels showed a worse overall survival rate (*P* = 0.007, Fig 4B).

### Univariate and multivariate analysis

To evaluate the role of clinicopathological parameters as probable risk factors for prognosis, univariate analysis was performed to analyze 13 clinicopathological variables in the 86 CCA patients. As shown in Table 2, the results indicated that tumor differentiation (*P* = 0.026), lymph node metastasis (*P* = 0.004), vascular invasion (*P <* 0.001), TNM stage (*P* = 0.008), SOCS3 expression (*P* = 0.001) and A20 expression (*P <* 0.001) were prognostic factors with statistical significance (Table 2). Then, the 6 risk factors filtered by the log-rank test were further analyzed by multivariate analysis to determine independent factors for prognosis. As presented in Table 3, only lymph node metastasis (*P* = 0.007; HR, 1.142), vascular invasion (*P* = 0.001; HR, 1.370), SOCS3 expression (*P* = 0.022; HR, 2.382) and A20 expression (*P* = 0.009; HR, 6.598) served as independent biomarkers for prognosis, indicating that from all 13 biomarkers, a low SOCS3 level and high A20 level may represent the best prognostic indicators of survival in CCA.

**Table 2:** Univariate log-rank test of clinicopathological features of 86 patients with cholangiocarcinoma.

Clinicopathologic features	No. patients	Survival rate (%)	*P*
**Age (years)**			
<60	37	57.1%	0.893
≥60	49	52.4%	
**Gender**			
Male	54	49.2%	0.312
Female	32	65.3%	
**Tumor site**			
Intrahepatic	18	31.1%	0.545
Perihilar	33	59.2%	
Distal	35	58.6%	
**Differentiation**			
Grade 1	16	68.8%	**0.026**
Grade 2	38	60.2%	
Grade 3	32	33.6%	
**Histological patterns**			
Tubular adenocarcinoma	76	54.2%	0.088
Papillary adenocarcinoma	4	50.0%	
Mcinous adenocarcinoma	6	25.0%	
**Lymph node metastasis**			
Positive	51	37.0%	**0.004**
Negative	35	68.6%	
**Vascular invasion**			
Positive	12	28.4%	**< 0.001**
Negative	74	57.8%	
**TNM stage**			
I	12	75.0%	**0.008**
II	23	63.6%	
III	32	49.0%	
IVA	19	30.2%	
**Serum CEA**			
>5 ng/ml	51	57.4%	0.774
≤5 ng/ml	35	52.6%	
**Serum CA199**			
>37 U/ml	56	53.2%	0.933
≤37 U/ml	30	56.6%	
**HBV infection**			
Positive	42	46.4%	0.544
Negative	44	62.9%	
**SOCS3 expression level**			
High	24	73.4%	**0.008**
Low	62	45.9%	
**A20 expression level**			
High	59	45.6%	**0.007**
Low	27	71.4%	

**Table 3:** Multivariate Cox regression analysis of clinicopathological features of 86 patients with cholangiocarcinoma.

Factors	Category	*P*	HR	95% CI
**Differentiation**	Grade 3	0.285	0.396	0.203–0.718
	Grade 1+ Grade 2			
**TNM stage**	III+ IVA	0.715	0.434	0.169–0.835
	I+ II			
**Vascular invasion**	Positive	0.001	1.370	1.251–1.642
	Negative			
**Lymph node metastasis**	Positive	0.007	1.142	1.036–1.437
	Negative			
**SOCS3 expression level**	Low	0.022	2.382	1.253–4.722
	High			
**A20 expression level**	High	0.009	6.598	2.584–14.261
	Low			

HR, hazard ratio; CI, confidence interval.

## Discussion

Although the downregulation of SOCS3 has been regarded as crucial in tumor proliferation and migration [40], few studies have assessed the prognostic role of SOCS3 in human CCA. In addition, to the best of our knowledge, little is known about the role of A20 in predicting CCA patients' prognosis. Herein, our study suggests that SOCS3 and A20 may serve as prognostic biomarkers in CCA, and our results support the hypothesis that A20 enhances JAK/STAT signaling in CCA by down-regulating SOCS3. Initially, we compared SOCS3 and A20 protein expression levels by western blot analysis in 22 cases of freshly frozen CCA tumors and their corresponding peritumoral biliary tissues. Compared with the corresponding peritumoral biliary tissues, the CCA tumor tissues showed remarkably higher A20 protein expression levels. However, lower SOCS3 protein expression was observed in CCA tumor tissues than the corresponding peritumoral biliary tissues. Linear regression analysis revealed that A20 levels were inversely correlated with SOCS3 expression (*R* ^*2*^ = 0.8232, *P <* 0.0001). Next, we examined the SOCS3 and A20 protein expression levels in 86 cases of surgical specimens using IHC and detected a correlation between SOCS3 and A20 expression. Our data demonstrated that there was an inverse relationship (r = -0.585, *P <* 0.0001) between the expression levels of SOCS3 and A20 in CCA. To investigate the role of prognostic biomarkers, we further analyzed the SOCS3 and A20 protein expression levels with corresponding clinicopathological features in 86 CCA cases. These results suggested that patients with low SOCS3 levels had a remarkably worse overall survival rate compared to those with high SOCS3 levels (*P* = 0.001, Fig 4A), whereas patients with high A20 levels had a worse overall survival rate than those with low A20 levels (*P <* 0.001, Fig 4B).

The JAK/STAT pathway is constitutively activated in various human malignancies, particularly in the tumor subtypes associated with chronic inflammation [41, 42], including CCA, as recently reported [43]. Activation of the JAK/STAT pathway results in tyrosine-phosphorylated STAT3, which is involved in many aspects of tumorigenesis, including proliferation, differentiation, apoptosis, modulation of sensitivity to cytotoxic agents, angiogenesis, recruitment of immune cells, and metastasis [9–12]. Decreased expression of SOCS proteins through promoter methylation significantly contributes to the persistent tyrosine phosphorylation of STAT3 in cancers [44], as well as in CCA [21].

Our recent studies found that high expression of SOCS3 could reduce tumor metastasis, EMT markers and STAT3 activation in the absence of IL-6 stimulation in CCA cell lines [22]. In this study, we reinforced the putative prognostic role of SOCS3 in CCA by western blot and IHC in human surgical specimens. Using western blot analysis, the SOCS3 signal was positive in only 27.27% (6 cases) of the freshly frozen CCA tumor tissues, while it was positive in all (22 cases) corresponding peritumoral biliary tissues. The loss of SOCS3 expression in malignancies has been extensively described [16–20], suggesting that it may serve as a general mechanism for activating the JAK/STAT pathway in solid tumors. We found that SOCS3 expression was decreased in 72.1% of the CCA specimens (62 cases) by IHC. Furthermore, we found that SOCS3 expression was remarkably correlated with lymph node invasion and histological type in CCA. Moreover, patients with low SOCS3 levels showed a poor overall survival rate (45.9% vs 73.4%), as evaluated by univariate analysis, and multivariate Cox analysis identified SOCS3 as an independent risk factor for prognosis (*P* = 0.022; HR, 2.382). What was most interesting was that SOCS3 repression was associated with A20 overexpression in CCA tissues.

A20 functions as a tumor enhancer due to its overexpression in multiple malignant solid tumors [25–30] and also as a ubiquitin-editing enzyme that regulates inflammatory responses and cell immunoreaction [23]. In lymphoma, however, A20 repression as well as mutation is commonly observed, which implies its role as a suppressor in tumor biology [32–35]. Therefore, whether A20 acts as an enhancer or a suppressor in tumor biology may depend on the tissue type and tumor stage [27]. According to the results of our western blot analysis, the A20 signal was positive in all (22 cases) freshly frozen CCA tumor tissues and in 68.75% (15 cases) of the corresponding peritumoral biliary tissues. Furthermore, the protein expression levels of A20 in CCA tumor tissues were remarkably higher compared to those in the corresponding peritumoral biliary tissues (2.23-fold on average, *P <* 0.0001) (Fig 1C). By IHC, high A20 expression was detected in 68.6% (59 cases) of the CCA specimens. Patients with high A20 expression also showed a poor overall survival rate (71.4% vs 45.6%), as evaluated by univariate analysis, and A20 was identified as an independent biomarker for prognosis by multivariate Cox analysis (*P* = 0.009; HR, 6.598). These results suggest that A20 may function as an enhancer in the tumor biology of CCA, which is consistent with previous studies of other malignant solid tumors, such as undifferentiated nasopharyngeal carcinoma, poorly differentiated head and neck squamous cell carcinoma [25], glioma [26, 27], glioblastoma [28], inflammatory breast cancer [29], and hepatocellular carcinoma [30], where A20 expression was related to an undesirable prognosis.

We further demonstrated that A20 expression in CCA was significantly associated with tumor differentiation, TNM stage and lymph node metastasis, and multivariate analysis confirmed A20 as an independent prognostic factor according to the Cox proportional hazard regression model. Interestingly, the inverse correlation between A20 overexpression and SOCS3 repression discovered in our study suggests that the mechanism of SOCS3 downregulation in CCA may be similar to that reported in a previous study of liver regeneration [24], which showed that A20 enhanced JAK/STAT signals in hepatocytes by downregulating SOCS3 in the process of liver regeneration. However, we noticed that 17.5% of the patients in this study did not fit into the category where the relationship between A20 and SOCS3 was inverse (Fig 3), and this result indicates that there might be alternative oncogenic pathways in CCA development for these patients. Moreover, little is known about the mutual impact of SOCS3 and A20 in CCA, and intensive investigation will be performed on this topic in our future study.

Most of the risk factors for CCA, including primary sclerosing cholangitis (PSC), chronic hepatolithiasis and choledocholithiasis, choledochal cysts, chronic hepatitis C virus (HCV) and parasitic bile duct infection, are immediate causes of chronic biliary inflammation. Anomalies in growth regulatory genes have been partially illuminated in established malignant tumors in several studies and may serve as the mechanism for the interaction between biliary chronic inflammation and carcinogenesis [45–47]. During chronic inflammation, activation of the JAK/STAT pathway plays a crucial role in tumor progression, not only in tumor cells but also dendritic cells, myeloid cells, and B and T cells, to establish a chronic inflammatory environment but also in epithelial cells to generate cytokines and chemokines [48]. Tumors originating in these environments maintain secretion of these inflammatory factors most likely because they supply tumor growth factors and favorable survival conditions.

Our early research revealed that IL-6, through activation of JAK/STAT signaling, is involved in the promotion of human biliary epithelial cell proliferation and migration [12]. Subsequently, our recent study confirmed SOCS3 as a negative feedback regulator, controlling the JAK/STAT pathway in relation to cell proliferation and migration through a classic feedback loop in human CCA cell lines [22]. Combined with our present study, these results show the significant role that SOCS3 plays in regulating JAK/STAT signaling during the process of inflammation in the development of CCA, thereby predicting the prognosis of CCA patients. Previous research has demonstrated that A20 regulates the JAK/STAT pathway by upregulating miR203 levels, which decreases SOCS3 mRNA expression. This decrease activates JAK/STAT3 signaling and the transcription of STAT3-dependent mitogenetic genes, such as cyclin A and cyclin D1, while blocking NF-κB signaling, thus decreasing transcription of STAT3-dependent proinflammatory genes [24]. Taken together, our data and previous studies [24] support the hypothesis that A20 enhances the activity of the JAK/STAT pathway in CCA by decreasing the SOCS3 level.

CCA is notorious for its devastating prognosis in advanced stages and the lack of conspicuous clinical features in early stages [49]; furthermore, the incidence of CCA is increasing worldwide [50]. Since this disease was first systematically described by Klatskin, advances have been made towards the diagnosis and treatment of CCA in recent decades. Nevertheless, the survival rate of CCA remains unsatisfactory, even for patients who undergo curative surgery [5]. Therefore, the identification of new cancerous biomarkers in surgical specimens is needed for promoting both early diagnosis and prognosis of CCA. This study is the first to provide evidence for the unanticipated interaction of the inflammatory regulators A20 and SOCS3 in CCA. Our data offer vital information for the prediction that A20 and SOCS3 could be used as a novel prognostic biomarkers in CCA and may be regarded as new therapeutic targets for future CCA treatment.

## Supporting Information

S1 Table The follow-up data of 86 CCA patients. (XLS)

## References

[1] Blechacz B, Gores GJ. Cholangiocarcinoma: advances in pathogenesis, diagnosis, and treatment. Hepatology. 2008;48(1):308–21. 10.1002/hep.22310 18536057 PMC2547491

[2] Palmer WC, Patel T. Are common factors involved in the pathogenesis of primary liver cancers? A meta-analysis of risk factors for intrahepatic cholangiocarcinoma. Journal of hepatology. 2012;57(1):69–76. 10.1016/j.jhep.2012.02.022 22420979 PMC3804834

[3] El Khatib M, Kalnytska A, Palagani V, Kossatz U, Manns MP, Malek NP, et al Inhibition of hedgehog signaling attenuates carcinogenesis in vitro and increases necrosis of cholangiocellular carcinoma. Hepatology. 2013;57(3):1035–45. 10.1002/hep.26147 . 23172661

[4] Kang P, Wan M, Huang P, Li C, Wang Z, Zhong X, et al The Wnt antagonist sFRP1 as a favorable prognosticator in human biliary tract carcinoma. PloS one. 2014;9(3):e90308 10.1371/journal.pone.0090308 24594839 PMC3940873

[5] Razumilava N, Gores GJ. Cholangiocarcinoma. The Lancet. 2014;383(9935):2168–79. 10.1016/s0140-6736(13)61903-0 PMC4069226 24581682

[6] Sia D, Hoshida Y, Villanueva A, Roayaie S, Ferrer J, Tabak B, et al Integrative molecular analysis of intrahepatic cholangiocarcinoma reveals 2 classes that have different outcomes. Gastroenterology. 2013;144(4):829–40. 10.1053/j.gastro.2013.01.001 23295441 PMC3624083

[7] Bergquist A, Ekbom A, Olsson R, Kornfeldt D, Loof L, Danielsson A, et al Hepatic and extrahepatic malignancies in primary sclerosing cholangitis. Journal of hepatology. 2002;36(3):321–7. . 11867174 10.1016/s0168-8278(01)00288-4

[8] Tocchi A, Mazzoni G, Liotta G, Lepre L, Cassini D, Miccini M. Late development of bile duct cancer in patients who had biliary-enteric drainage for benign disease: a follow-up study of more than 1,000 patients. Annals of surgery. 2001;234(2):210–4. 11505067 10.1097/00000658-200108000-00011 PMC1422008

[9] Isomoto H, Kobayashi S, Werneburg NW, Bronk SF, Guicciardi ME, Frank DA, et al Interleukin 6 upregulates myeloid cell leukemia-1 expression through a STAT3 pathway in cholangiocarcinoma cells. Hepatology. 2005;42(6):1329–38. 10.1002/hep.20966 . 16317687

[10] Hodge DR, Hurt EM, Farrar WL. The role of IL-6 and STAT3 in inflammation and cancer. European journal of cancer. 2005;41(16):2502–12. 10.1016/j.ejca.2005.08.016 . 16199153

[11] Mertens C, Darnell JE Jr. SnapShot: JAK-STAT signaling. Cell. 2007;131(3):612 10.1016/j.cell.2007.10.033 . 17981126

[12] Jiang GX, Zhong XY, Cui YF, Liu W, Tai S, Wang ZD, et al IL-6/STAT3/TFF3 signaling regulates human biliary epithelial cell migration and wound healing in vitro. Molecular biology reports. 2010;37(8):3813–8. 10.1007/s11033-010-0036-z . 20229017

[13] Aaronson DS, Horvath CM. A road map for those who don't know JAK-STAT. Science. 2002;296(5573):1653–5. 10.1126/science.1071545 . 12040185

[14] O'Shea JJ, Gadina M, Schreiber RD. Cytokine signaling in 2002: new surprises in the Jak/Stat pathway. Cell. 2002;109 Suppl:S121–31. . 11983158 10.1016/s0092-8674(02)00701-8

[15] Park EJ, Park SY, Joe EH, Jou I. 15d-PGJ2 and rosiglitazone suppress Janus kinase-STAT inflammatory signaling through induction of suppressor of cytokine signaling 1 (SOCS1) and SOCS3 in glia. The Journal of biological chemistry. 2003;278(17):14747–52. 10.1074/jbc.M210819200 . 12584205

[16] Kim MH, Kim MS, Kim W, Kang MA, Cacalano NA, Kang SB, et al Suppressor of cytokine signaling (SOCS) genes are silenced by DNA hypermethylation and histone deacetylation and regulate response to radiotherapy in cervical cancer cells. PloS one. 2015;10(4):e0123133 10.1371/journal.pone.0123133 25849377 PMC4388447

[17] He B, You L, Uematsu K, Zang K, Xu Z, Lee AY, et al SOCS-3 is frequently silenced by hypermethylation and suppresses cell growth in human lung cancer. Proceedings of the National Academy of Sciences of the United States of America. 2003;100(24):14133–8. 10.1073/pnas.2232790100 14617776 PMC283558

[18] Feng Y, Wang Z, Bao Z, Yan W, You G, Wang Y, et al SOCS3 promoter hypermethylation is a favorable prognosticator and a novel indicator for G-CIMP-positive GBM patients. PloS one. 2014;9(3):e91829 10.1371/journal.pone.0091829 24633048 PMC3954800

[19] Sandoval-Usme MC, Umana-Perez A, Guerra B, Hernandez-Perera O, Garcia-Castellano JM, Fernandez-Perez L, et al Simvastatin impairs growth hormone-activated signal transducer and activator of transcription (STAT) signaling pathway in UMR-106 osteosarcoma cells. PloS one. 2014;9(1):e87769 10.1371/journal.pone.0087769 24489959 PMC3906206

[20] Rossa C Jr, Sommer G, Spolidorio LC, Rosenzweig SA, Watson DK, Kirkwood KL. Loss of expression and function of SOCS3 is an early event in HNSCC: altered subcellular localization as a possible mechanism involved in proliferation, migration and invasion. PloS one. 2012;7(9):e45197 10.1371/journal.pone.0045197 23028842 PMC3445460

[21] Isomoto H, Mott JL, Kobayashi S, Werneburg NW, Bronk SF, Haan S, et al Sustained IL-6/STAT-3 signaling in cholangiocarcinoma cells due to SOCS-3 epigenetic silencing. Gastroenterology. 2007;132(1):384–96. 10.1053/j.gastro.2006.10.037 17241887 PMC2203612

[22] Zhou QX, Jiang XM, Wang ZD, Li CL, Cui YF. Enhanced expression of suppresser of cytokine signaling 3 inhibits the IL-6-induced epithelial-to-mesenchymal transition and cholangiocarcinoma cell metastasis. Medical oncology. 2015;32(4):105 10.1007/s12032-015-0553-7 . 25744243

[23] Verstrepen L, Verhelst K, van Loo G, Carpentier I, Ley SC, Beyaert R. Expression, biological activities and mechanisms of action of A20 (TNFAIP3). Biochemical pharmacology. 2010;80(12):2009–20. . 20599425 10.1016/j.bcp.2010.06.044

[24] da Silva CG, Studer P, Skroch M, Mahiou J, Minussi DC, Peterson CR, et al A20 promotes liver regeneration by decreasing SOCS3 expression to enhance IL-6/STAT3 proliferative signals. Hepatology. 2013;57(5):2014–25. 10.1002/hep.26197 23238769 PMC3626749

[25] Codd JD, Salisbury JR, Packham G, Nicholson LJ. A20 RNA expression is associated with undifferentiated nasopharyngeal carcinoma and poorly differentiated head and neck squamous cell carcinoma. The Journal of pathology. 1999;187(5):549–55. 10.1002/(SICI)1096-9896(199904)187:5<549::AID-PATH278>3.0.CO;2-O . 10398120

[26] Guo Q, Dong H, Liu X, Wang C, Liu N, Zhang J, et al A20 is overexpressed in glioma cells and may serve as a potential therapeutic target. Expert opinion on therapeutic targets. 2009;13(7):733–41. 10.1517/14728220903045018 . 19492975

[27] Hjelmeland AB, Wu Q, Wickman S, Eyler C, Heddleston J, Shi Q, et al Targeting A20 decreases glioma stem cell survival and tumor growth. PLoS biology. 2010;8(2):e1000319 10.1371/journal.pbio.1000319 20186265 PMC2826371

[28] Verbrugge I, Johnstone RW. Regulating the TRAIL of destruction: how A20 protects glioblastomas from TRAIL-mediated death. Cancer discovery. 2012;2(2):112–4. 10.1158/2159-8290.CD-11-0350 . 22585854

[29] Lerebours F, Vacher S, Andrieu C, Espie M, Marty M, Lidereau R, et al NF-kappa B genes have a major role in inflammatory breast cancer. BMC cancer. 2008;8:41 10.1186/1471-2407-8-41 18248671 PMC2267801

[30] Dong B, Lv G, Wang Q, Wei F, Bellail AC, Hao C, et al Targeting A20 enhances TRAIL-induced apoptosis in hepatocellular carcinoma cells. Biochemical and biophysical research communications. 2012;418(2):433–8. 10.1016/j.bbrc.2012.01.056 . 22285182

[31] Vendrell JA, Ghayad S, Ben-Larbi S, Dumontet C, Mechti N, Cohen PA. A20/TNFAIP3, a new estrogen-regulated gene that confers tamoxifen resistance in breast cancer cells. Oncogene. 2007;26(32):4656–67. 10.1038/sj.onc.1210269 . 17297453

[32] Compagno M, Lim WK, Grunn A, Nandula SV, Brahmachary M, Shen Q, et al Mutations of multiple genes cause deregulation of NF-kappaB in diffuse large B-cell lymphoma. Nature. 2009;459(7247):717–21. 10.1038/nature07968 19412164 PMC2973325

[33] Kato M, Sanada M, Kato I, Sato Y, Takita J, Takeuchi K, et al Frequent inactivation of A20 in B-cell lymphomas. Nature. 2009;459(7247):712–6. 10.1038/nature07969 . 19412163

[34] Honma K, Tsuzuki S, Nakagawa M, Tagawa H, Nakamura S, Morishima Y, et al TNFAIP3/A20 functions as a novel tumor suppressor gene in several subtypes of non-Hodgkin lymphomas. Blood. 2009;114(12):2467–75. 10.1182/blood-2008-12-194852 . 19608751

[35] Schmitz R, Hansmann ML, Bohle V, Martin-Subero JI, Hartmann S, Mechtersheimer G, et al TNFAIP3 (A20) is a tumor suppressor gene in Hodgkin lymphoma and primary mediastinal B cell lymphoma. The Journal of experimental medicine. 2009;206(5):981–9. 10.1084/jem.20090528 19380639 PMC2715030

[36] Liu J, Wang Y, Du W, Liu W, Liu F, Zhang L, et al Wnt1 inhibits hydrogen peroxide-induced apoptosis in mouse cardiac stem cells. PloS one. 2013;8(3):e58883 10.1371/journal.pone.0058883 23533594 PMC3606408

[37] Ding BS, Cao Z, Lis R, Nolan DJ, Guo P, Simons M, et al Divergent angiocrine signals from vascular niche balance liver regeneration and fibrosis. Nature. 2014;505(7481):97–102. 10.1038/nature12681 24256728 PMC4142699

[38] Kim KH, Chen CC, Alpini G, Lau LF. CCN1 induces hepatic ductular reaction through integrin alphavbeta(5)-mediated activation of NF-kappaB. The Journal of clinical investigation. 2015;125(5):1886–900. 10.1172/JCI79327 25822023 PMC4463205

[39] Hao XP, Willis JE, Pretlow TG, Rao JS, MacLennan GT, Talbot IC, et al Loss of fragile histidine triad expression in colorectal carcinomas and premalignant lesions. Cancer research. 2000;60(1):18–21. . 10646844

[40] Culig Z. Suppressors of cytokine signalling-3 and -1 in human carcinogenesis. Frontiers in bioscience. 2013;5:277–83. . 23277051 10.2741/s372

[41] Grivennikov S, Karin E, Terzic J, Mucida D, Yu GY, Vallabhapurapu S, et al IL-6 and Stat3 are required for survival of intestinal epithelial cells and development of colitis-associated cancer. Cancer cell. 2009;15(2):103–13. 10.1016/j.ccr.2009.01.001 19185845 PMC2667107

[42] Yu H, Pardoll D, Jove R. STATs in cancer inflammation and immunity: a leading role for STAT3. Nature reviews Cancer. 2009;9(11):798–809. 10.1038/nrc2734 . 19851315 PMC4856025

[43] Zheng T, Hong X, Wang J, Pei T, Liang Y, Yin D, et al Gankyrin promotes tumor growth and metastasis through activation of IL-6/STAT3 signaling in human cholangiocarcinoma. Hepatology. 2014;59(3):935–46. 10.1002/hep.26705 . 24037855

[44] Sansone P, Bromberg J. Targeting the interleukin-6/Jak/stat pathway in human malignancies. Journal of clinical oncology: official journal of the American Society of Clinical Oncology. 2012;30(9):1005–14. 10.1200/JCO.2010.31.8907 22355058 PMC3341105

[45] Chapman MH, Webster GJ, Bannoo S, Johnson GJ, Wittmann J, Pereira SP. Cholangiocarcinoma and dominant strictures in patients with primary sclerosing cholangitis: a 25-year single-centre experience. European journal of gastroenterology & hepatology. 2012;24(9):1051–8. 10.1097/MEG.0b013e3283554bbf 22653260 PMC3584158

[46] Broome U, Olsson R, Loof L, Bodemar G, Hultcrantz R, Danielsson A, et al Natural history and prognostic factors in 305 Swedish patients with primary sclerosing cholangitis. Gut. 1996;38(4):610–5. 8707097 10.1136/gut.38.4.610 PMC1383124

[47] Bergquist A, Broome U. Hepatobiliary and extra-hepatic malignancies in primary sclerosing cholangitis. Best practice & research Clinical gastroenterology. 2001;15(4):643–56. 10.1053/bega.2001.0210 . 11492973

[48] Moss SF, Blaser MJ. Mechanisms of disease: Inflammation and the origins of cancer. Nature clinical practice Oncology. 2005;2(2):90–7; quiz 1 p following 113. 10.1038/ncponc0081 . 16264881

[49] Khan SA, Thomas HC, Davidson BR, Taylor-Robinson SD. Cholangiocarcinoma. The Lancet. 2005;366(9493):1303–14. 10.1016/s0140-6736(05)67530-7 16214602

[50] Malhi H, Gores GJ. Cholangiocarcinoma: modern advances in understanding a deadly old disease. Journal of hepatology. 2006;45(6):856–67. 10.1016/j.jhep.2006.09.001 17030071 PMC1686172

